# A simple method for sequencing the whole human mitochondrial genome directly from samples and its application to genetic testing

**DOI:** 10.1038/s41598-019-53449-y

**Published:** 2019-11-22

**Authors:** Yue Yao, Motoi Nishimura, Kei Murayama, Naomi Kuranobu, Satomi Tojo, Minako Beppu, Takayuki Ishige, Sakae Itoga, Sachio Tsuchida, Masato Mori, Masaki Takayanagi, Masataka Yokoyama, Kazuyuki Yamagata, Yoshihito Kishita, Yasushi Okazaki, Fumio Nomura, Kazuyuki Matsushita, Tomoaki Tanaka

**Affiliations:** 1 Department of Molecular Diagnosis, Graduate School of Medicine, Chiba University, 1-8-1 Inohana, Chuo-ku, Chiba 260-8670 Japan; 2 Division of Laboratory Medicine, Clinical Genetics and Proteomics, Chiba University Hospital, 1-8-1 Inohana, Chuo-ku, Chiba 260-8670 Japan; 3 Division of Metabolism, Chiba Children’s Hospital, Chiba, 266-0007 Japan; 4 Division of Clinical Mass Spectrometry, Chiba University Hospital, 1-8-1 Inohana, Chuo-ku, Chiba, 260-8670 Japan; 5 Department of Pediatrics, Matsudo City Hospital, Matsudo, 270-2296 Japan; 6 Diagnostics and Therapeutics of Intractable Diseases, Intractable Disease Research Center, Graduate School of Medicine, Juntendo University, Hongo 2-1-1, Bunkyo-ku, Tokyo 113-8421 Japan

**Keywords:** Diagnostic markers, Molecular medicine

## Abstract

Next-generation sequencing (NGS) is a revolutionary sequencing technology for analyzing genomes. However, preprocessing methods for mitochondrial DNA (mtDNA) sequencing remain complex, and it is required to develop an authenticated preprocessing method. Here, we developed a simple and easy preprocessing method based on isothermal rolling circle mtDNA amplification using commercially available reagents. Isothermal amplification of mtDNA was successfully performed using both nanoliter quantities of plasma directly and 25 ng of total DNA extracted from blood or tissue samples. Prior to mtDNA amplification, it was necessary to treat the extracted total DNA with Exonuclease V, but it was not required to treat plasma. The NGS libraries generated from the amplified mtDNA provided sequencing coverage of the entire human mitochondrial genome. Furthermore, the sequencing results successfully detected heteroplasmy in patient samples, with called mutations and variants matching those from previous, independent, Sanger sequencing analysis. Additionally, a novel single nucleotide variant was detected in a healthy volunteer. The successful analysis of mtDNA using very small samples from patients is likely to be valuable in clinical medicine, as it could reduce patient discomfort by reducing sampling-associated damage to tissues. Overall, the simple and convenient preprocessing method described herein may facilitate the future development of NGS-based clinical and forensic mtDNA tests.

## Introduction

Next-generation sequencing (NGS) is a revolutionary^1^ sequencing technology for analyzing genomes or transcripts on a large scale and is currently being used in many research fields. Three major NGS sequencing platforms (Ion Torrent Personal Genome Machine PGM, Pacific Biosciences RS, and Illumina MiSeq) allow whole exome analyses of small genomes in short periods of time and at low cost^2,3^.

Human mitochondrial DNA (mtDNA), located in mitochondria, comprises 16,569 base pairs, including a forensically valuable polymorphic region, and encodes the mitochondrial 16 S and 12 S ribosomal RNAs, 22 mitochondrial tRNAs, and 13 respiratory chain proteins^4^. Generally, mtDNA is only inherited from the mother.

Mutations in mtDNA are an important cause of inherited diseases, as is evident from the morbid map of mtDNA (Mitomap, https://www.mitomap.org/MITOMAP). Furthermore, the relatively high mtDNA mutation rate makes haplogroup determination and classification an important tool for paleoanthropology, population genetics, and forensic medicine^5,6^. However, challenges remain for the prevention and treatment of mtDNA-associated diseases (e.g., diabetes mellitus and Leber’s hereditary optic neuropathy)^7–9^.

Accurate mtDNA analysis is needed to understand the relationship between mtDNA sequence and disease phenotypes. NGS platforms allow dramatically faster, higher throughput, and more cost-effective sequencing compared with traditional capillary sequencing^10^. However, the accuracy of NGS analysis is affected by factors such as GC bias^11^ and nuclear mitochondrial DNA sequences (Numts), which can vary in sequence and copy number^12–14^.

Previously, several new analysis methods were established to address these problems, including long range polymerase chain reaction (PCR) using mtDNA-specific primers^15^, whole genome panels resulting in small amplicons for amplifying and sequencing the mtDNA^16^, capture-based methods for sequencing mtDNA^17^, and DNA digestion with methylation-specific endonucleases MspJI and AbaSI to deplete nuclear DNA (nDNA) that is likely to be methylated^18–20^. Among these NGS pretreatment methods, long range PCR method has the advantage to allow the mtDNA analysis for small amount of samples, therefore this method is widely used for genetic testing even it can involve an indivisable amplification precess. The properties of the mitochondrial genome have been utilized recently to develop several new pretreatment methods for mitochondrial genome analysis, such as Mseek^21^ and MitoRS (mitochondrial DNA analysis by Rolling circle amplification [RCA] and Sequencing)^22,23^. In recent days, isothermal amplification methods have been applied to genetic testing gradually^24–26^. Since isothermal amplification methods can amplify DNA at a constant temperature, the reaction conditions are simpler than the conventional PCR method, and a thermal cycler is not required for DNA amplification. Herein, to offer a novel complementary tool to the long-range PCR method, we combined the Ion Torrent PGM, a benchtop NGS system, with ready-made, commercially available reagents to develop a simple and easy preprocessing method for mtDNA NGS analysis from a small amount of sample. We then applied this method to identify mtDNA variants in the Japanese population.

## Materials and Methods

### mtDNA sources and ethics

Our preprocessing method was developed using samples obtained from three healthy volunteers at Chiba University, Japan. We then evaluated and validated the method using samples from pediatric patients with mitochondrial disease provided by Chiba Children’s Hospital; the mtDNA in these samples, comprising one heart sample, 14 muscle samples, and one liver sample (Supplementary Table 1), had previously been analyzed using standard Sanger sequencing. Finally, we applied the method using samples taken from 20 healthy volunteers at Chiba University to identify mtDNA variants in the Japanese population.

Written informed consent was obtained prior to all sample collection. Informed consent was obtained from a parent of participants when they are under the age of 18 years. The genetic analyses were approved by the Human Ethics Committee of Chiba University or the Human Ethics Committee of Chiba Children’s Hospital. The methods were carried out in accordance with the approved guidelines and the Declaration of Helsinki.

### Sample treatment and DNA extraction

Plasma samples were repeatedly frozen in liquid nitrogen and thawed a total of 10 times before mtDNA amplification in the presence of the anticoagulant EDTA. Total DNA was extracted from whole blood using the QIAamp DNA Blood Mini Kit (Qiagen, Hilden, Germany), and the extracted DNA concentration was calculated from the absorbance at 260 nm. Extracted total DNA was treated with Exonuclease V (New England Biolabs = NEB, Ipswich, MA, U.S.A.) following the manufacturer’s recommendations, and the digested DNA products were purified using Agencourt AMPure XP (Beckman, Brea, CA, U.S.A.) prior to mtDNA amplification.

### RCA-based mtDNA isothermal amplification

With plasma or extracted total DNA as template, mtDNA was amplified using a REPLI-g mitochondrial DNA kit (Qiagen) in strict accordance with the manufacturer’s recommendations. According to Qiagen’s manufacturer’s instruction and information, there is no risk of lack of mitochondrial DNA amplification due to a change in the primer binding site (i.e., caused by a deletion). The reason being the use of several primers in the amplification reaction with the REPLI-g Mitochondrial DNA Kit (as described on the company’s webpage, https://www.qiagen.com/).

Amplified mtDNA was treated with EcoRI (Takara, Kyoto, Japan), and the digested mtDNA was subjected to electrophoresis to assess if amplification was successful (Supplementary Fig. 1).

### Construction of NGS libraries

Following confirmation of mtDNA amplification, 100 ng of the amplified mtDNA was used to generate a sequencing library with NEBNext® Fast DNA Fragmentation & Library Prep Set for Ion Torrent ™ (NEB), strictly following the manufacturer’s recommendations. The steps for library construction were as follows: (i) fragmentation of mtDNA to 100–400 bp; (ii) adapter ligation and, if sequencing a large number of specimens in one run, differentiation of specimens using an Ion Xpress Barcode Adapters 1–16 Kit (Thermo Fisher Scientific, Waltham, MA, U.S.A.); (iii) purification of adapter ligated DNA using Agencourt AMPure XP (Beckman); (iv) selection for 200-bp fragments using E-Gel® SizeSelect ™ gels (Thermo Fisher Scientific); (v) amplification by PCR; and (vi) purification of the amplified library using Agencourt AMPure XP.

The concentration of the completed library was measured using a Agilent 2100 bioanalyzer and a High Sensitivity DNA Kit (Agilent, Santa Clara, CA, U.S.A.).

### Preparation of Ion PGM template

Ion Sphere Particle enrichment of the library and multiplex PCR were performed using an Ion PGM Template OT2 200 Kit (Thermo Fisher Scientific) supplied with the Ion OneTouch 2 system. Per the manufacturer’s recommendations, 6.5 µl library at a concentration of 100 pM was used for amplification.

### Sequencing and analysis

We conducted sequencing using an Ion PGM Sequencing 200 Kit v2 (Thermo Fisher Scientific) supplied with the Ion PGM system, strictly following the manufacturer’s recommendations.

To confirm the accuracy of the mutation calls, the Ion Torrent BAM files were analyzed using CLC Genomics Workbench (Qiagen) (CLC bio, Aarhus, Denmark) with default parameters and minimum thresholds for coverage and frequency of 50 and 5.0%, respectively. The mtDNA sequence used for reference was NC_012920.1 (GenBank RefSeq database, https://www.ncbi.nlm.nih.gov/nuccore/251831106). With reference to the historical database of Disease Mutations and General Variants described in Mitomap^27^, https://www.mitomap.org/MITOMAP), we classified the detected variation calls as being pathogenic or non-pathogenic.

The heteroplasmy rates of the m.3243 A > G and m.13513 G > A variations were independently analyzed by Polymerase Chain Reaction-Restriction Fragment Length Polymorphism (PCR-RFLP) as described previously^28^.

## Results

### Establishment of an experimental method for performing whole mtDNA sequencing analysis

**Figure 1:**
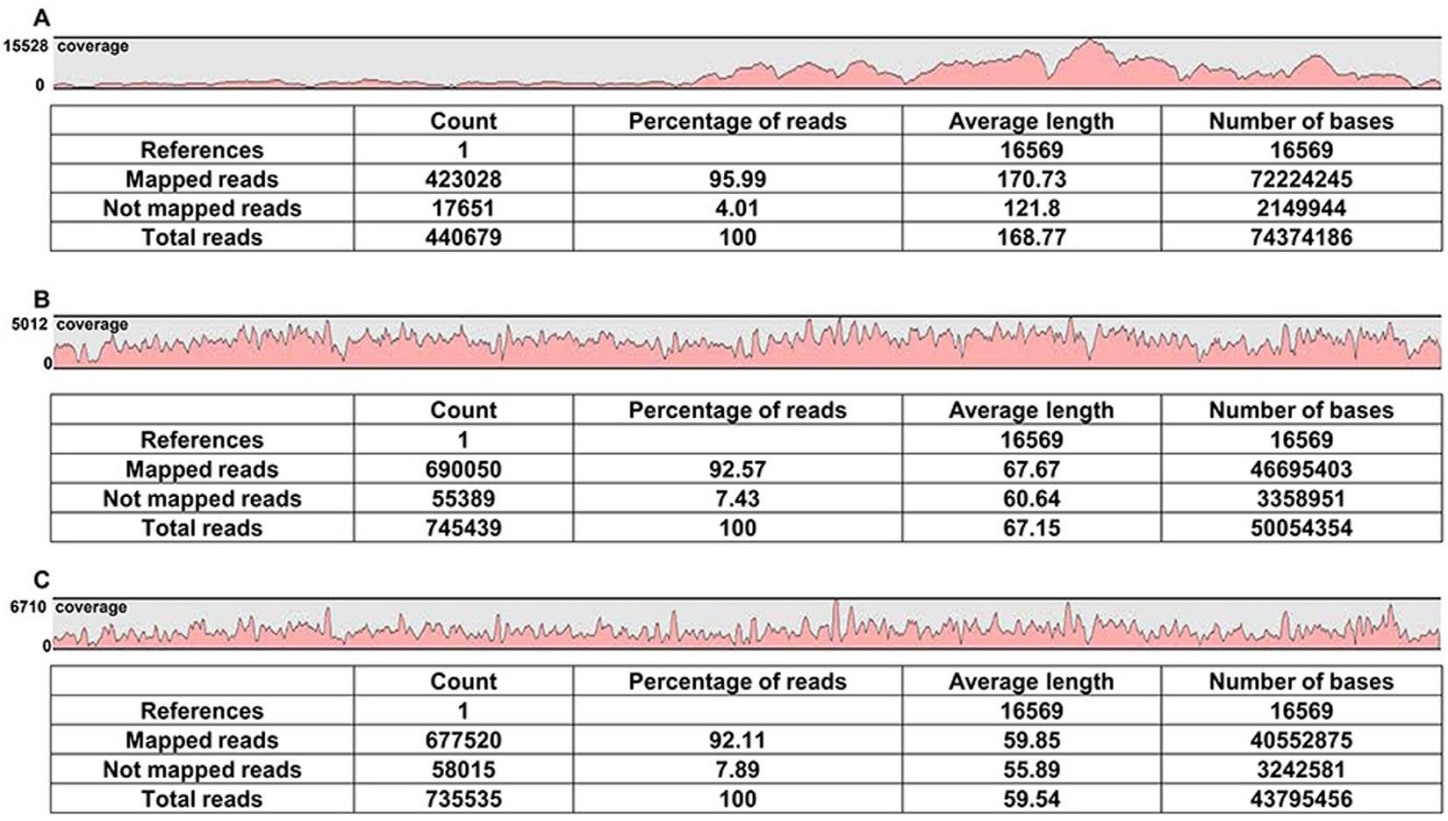
Mapping and coverage results for whole mtDNA sequencing of three samples from volunteer A. (**A–C**) Sequencing results for mtDNA amplified either directly from 200 nl plasma (**A**), or from 25 ng (**B**) or 2 µg (**C**) total DNA extracted from whole blood. The sequencing coverage for each sample is indicated in red. The maximum coverage for plasma, 25 ng total DNA, and 2 µg total DNA was 15228 reads, 5012 reads, and 6710 reads, respectively. All results were calculated using CLC Genomics Workbench (Qiagen).

The sequencing results from three samples (200 nl of plasma, 25 ng total DNA, and 2 µg total DNA) from a healthy subject called volunteer A were identical, and heteroplasmy was clearly evident (Table 1, Supplementary Fig. 2). The sequencing results for plasma samples from the other two volunteers (B and C) were also successfully completed (Supplementary Table 2). Isothermal amplification directly using plasma amplified sequences spanning most of the mtDNA with coverage of 50 reads at minimum, and as many as 95.99% of the reads mapped to mitochondrial sequence (Fig. 1A, Supplementary Fig. 3). When using total DNA isolated from whole blood, many sequences other than mtDNA tended to be amplified. Specifically, sequence reads of 24.61%, 21.32%, 2.47% and 8.61% from total DNA of volunteers (A, B and two other volunteers, respectively) were mapped to mitochondrial sequence. However, treatment of total DNA with Exonuclease V enabled specific amplification of mtDNA sequences, and sufficient coverage was obtained, with 92.57% or 92.11% of reads being successfully mapped when amplification was performed using 25 ng or 2 µg total DNA, respectively (Fig. 1B,C).

**Table 1:** SNVs called in whole mtDNA sequencing analysis of samples from volunteer A. Heteroplasmic positions are shown in red.

Position	Reference allele	Alternative allele	heteroplasmy/homoplasmy
73	A	G	Homoplasmy
150	C	T	Homoplasmy
263	A	G	Homoplasmy
709	G	A	Homoplasmy
750	A	G	Homoplasmy
1438	A	G	Homoplasmy
2706	A	G	Homoplasmy
3729	A	G	Homoplasmy
4769	A	G	Homoplasmy
5231	G	A	Homoplasmy
5417	G	A	Homoplasmy
5498	A	G	Homoplasmy
6915	G	A	Heteroplasmy
6915	G	G	Heteroplasmy
7028	C	T	Homoplasmy
8860	A	G	Homoplasmy
11719	G	A	Homoplasmy
12358	A	G	Homoplasmy
12372	G	A	Homoplasmy
12705	C	T	Homoplasmy
14766	C	T	Homoplasmy
15326	A	G	Homoplasmy
15883	G	A	Homoplasmy
16209	T	C	Homoplasmy
16223	C	T	Homoplasmy
16257	C	A	Homoplasmy
16261	C	T	Homoplasmy

The results from sequencing 200 nl plasma, 25 ng total DNA extracted from whole blood, and 2 ug total DNA extracted from whole blood matched completely.

Therefore we could detect heteroplasmy using this method in a small amount of total DNA (25 ng), indicating that this new preprocessing method could be a useful experimental protocol.

### RCA-based mtDNA amplification successfully identified mtDNA variants in pediatric patients with mitochondrial diseases, including two novel variants

**Figure 2:**
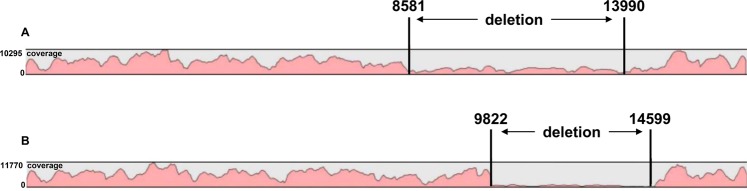
Mapping and coverage results for sequencing the mtDNA in two pediatric patients with mitochondrial disease, indicating deletion-type mutations. (**A,B**) Sequencing results for mtDNA indicating a deletion-type mutation between mtDNA nucleotides 8581 and 13990 in sample M132 (**A**) and a deletion-type mutation between mtDNA nucleotides 9822 and 14559 in sample Hep575 (**B**). The sequencing coverage is indicated in red. Additional information for both samples is provided in Supplemental Table 1.

**Figure 3:**
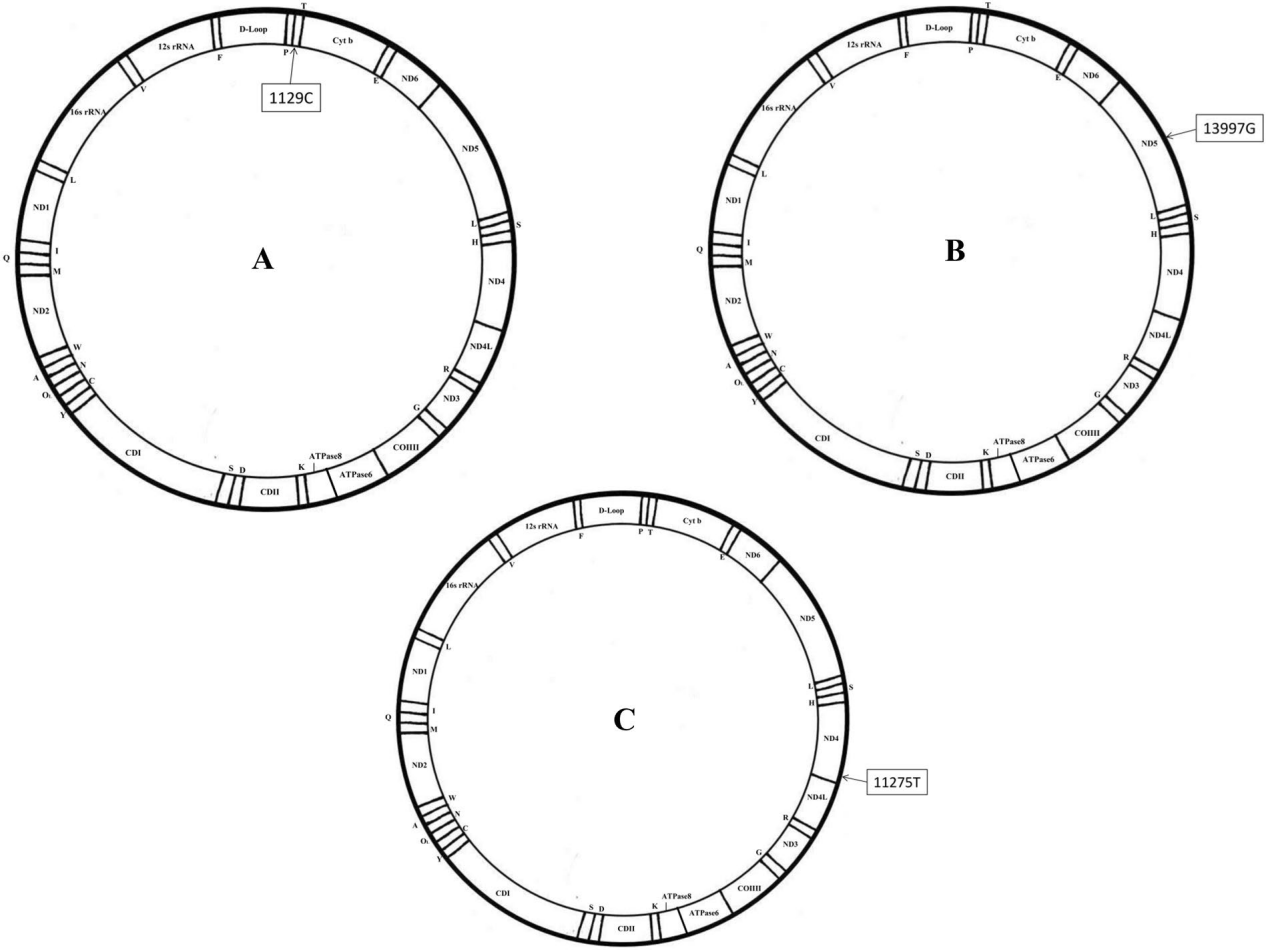
mtDNA morbid maps of novel variants identified in this study (**A**) mtDNA morbid map of SNV1129T > C in sample M52. The variation is heterozygous and located in the MT-RNR1 area of mtDNA and is related to 12 S ribosomal RNA. (**B**) mtDNA morbid map of SNV13997A > G in sample M132. The variation is heterozygous and located in the MT-ND5 area of mtDNA and is related to Nicotinamide adenine dinucleotide (NADH) dehydrogenase subunit 5. (**C**) mtDNA morbid map of SNV11275C > T in sample 079 C. The variation is homozygous and located in the MT-ND4 area of mtDNA and is related to Nicotinamide adenine dinucleotide (NADH) dehydrogenase subunit 4. Additional information for samples M52 and M132 is provided in Supplemental Table 1.

Our analyses of mtDNA in pediatric patients with mitochondrial diseases identified both pathogenic mutations and polymorphisms (Table 2, Figs 2 and 3, Supplementary Tables 3 and 4). These mutation calls agreed with the previous Sanger sequencing-based results at Chiba Children’s Hospital.

**Table 2:** Novel mtDNA variants identified in pediatric patients with mitochondrial disease.

Sample ID	Position	Reference allele	Alternative allele	Total reads	Allele frequency (%)	heteroplasmy/homoplasmy	Locus
M52	1129	T	C	5069	18.94	Heteroplasmy	MT-RNR1
			T		81.06		
M132	13997	A	A	1680	48.05	Heteroplasmy	MT-ND5
			G		51.95		

Heteroplasmic positions are shown in red. These novel variants have been registered in EMPOP (THE EUROPEAN DNA PROFILING GROUP = EDNAP mitochondrial DNA population database) (https://empop.online/) (Dataset EMP00730).

With reference to the historical data described in Mitomap, we classified the detected variation calls as being pathogenic (Supplementary Table 3) or non-pathogenic (Supplementary Table 4), with the latter including variations of unknown significance or low frequent polymorphisms. Among pathogenic variations, m.3243 A > G and m.13513 G > kA variations had their rates of heteroplasmy additionally quantified with an independent method (PCR-RFLP analysis), and the PCR-RFLP analysis demonstrates a reasonable result which is consistent with our RCA-based analysis (Supplementary Fig. 4). Additionally, Table 2 and Fig. 3A,B show novel variants which are not found in Mitomap. Separately, our analyses also identified two 5000-bp deletions (Fig. 2).

### RCA-based mtDNA amplification successfully identified control region variants (16024–576) and revealed a novel variant within the mtDNA coding region in a Japanese volunteer

Upon sequencing the mtDNA in 20 healthy volunteers, we compiled the identified variants and compared them to those present in Mitomap. The mtDNA control region (16024–576) was successfully sequenced (Supplementary Table 5), and we deteced several variants that were not novel but had known pathological roles were detected (Supplementary Table 6)^29^. Furthermore, a novel variant in the coding region was detected in one healthy volunteer (Fig. 3C, Table 3). mtDNA variations in pediatric patients and the volunteers are summarized in Supplementary Fig. 5 with ratios of the transition/transversion ratios, synonymous vs non-synonymous variations across almost all samples (in patients and volunteers).

**Table 3:** Novel mtDNA variant identified in one healthy volunteer.

Sample ID	Position	Reference allele	Alternative allele	Total reads	heteroplasmy/homoplasmy (Allele frequency)	Locus
079 C	11275	C	T	5664	Homoplasmy (100%)	MT-ND4

This novel variant has been registered in EMPOP (THE EUROPEAN DNA PROFILING GROUP = EDNAP mitochondrial DNA population database) (https://empop.online/) (Dataset EMP00730).

## Discussion

We successfully amplified mtDNA using an isothermal amplification method and sequenced whole mtDNA using small amounts of plasma directly or total DNA extracted from whole blood or muscle (Table 1, Fig. 1). Using this approach, we analyzed the mtDNA in pediatric patients with mitochondrial disease from samples provided by Chiba Children’s Hospital. The mutation calls identified herein for both pathogenic and non-pathogenic variations and polymorphisms agreed with the previous results separately obtained at Chiba Children’s Hospital (Table 2, Fig. 2, Supplementary Tables 3 and 4). Furthermore, mtDNA control regions (16024–576), which are valuable in paleoanthropology, population genetics, and forensic medicine, were successfully sequenced (Supplementary Table 5). By comparing our results with Mitomap variant data, a novel single nucleotide variant (SNV) in a healthy Japanese volunteer was detected (Fig. 3C, Table 3).

Using our new method, we achieved whole mtDNA sequencing in as little as 25 ng of total DNA. The sequencing result of 25 ng total DNA completely matched both 200nl of plasma and 2ug of total DNA in the same healthy volunteer (Fig. 1). There was no discernible interference from Numts using this method. Indeed, when starting from plasma or Exonuclease V-treated total DNA, we did not experience interference from nuclear DNA in our sequencing of the whole human mitochondrial genome.

Our results indicate that the total amount of DNA required for the analysis of mtDNA in pediatric patients with mitochondrial disease can be reduced to 25 ng. Moreover, the successful detection of heteroplasmic variations (Table 2, Supplementary Table 3) could improve individual identification in forensic or populational genetic studies. Previous forensic research has reported the detection of mtDNA heteroplasmy to be useful in establishing the authenticity of post-mortem remains after long periods of time, with the famous case of Tsar Nicholas II^30^. Still according to published literature, the benefits of whole mtDNA analysis in forensic DNA casework are considered of notable practicability^31^. The successful analysis of mtDNA using very small amounts of specimens or crude samples can be valuable to the fields of anthropology, population genetics, and forensic medicine, as it brings new possibilities to the table, such as reference samples from distant relatives or remains, which are often limited in quantity; therefore methods including an indivisable amplification process (i.e. long range PCR as well as our present RCA-based method) have the advantage over other NGS methods.

Furthermore, mitochondrial diseases associated with mtDNA mutations and its phenotypic heterogeneity can be explained by mtDNA heteroplasmy—the mixture of more than one type of mtDNA at a cellular, tissue or organism level^32^, it is not unusual that in diseased organ obvious heteroplasmy is found whereas in samples from peripheral tissue it is difficult to be reliably detected. Therefore, in many cases mtDNA from the damaged organ is needed to be tested for diagnosis.

The comparison of mtDNA variants identified herein with variant data in Mitomap resulted in the identification of a novel synonymous (glycine to glycine) SNV (11275 C > T) in a healthy volunteer. Since this SNV was identified in an apparently healthy individual, this SNV has no evident pathological significance. This SNV is homozygous and is located in the MT-ND4 region of the mtDNA, an area related to Nicotinamide adenine dinucleotide (NADH) dehydrogenase subunit 4 (Fig. 3C). This novel SNV may be specific to the Japanese population, but this possibility needs to be confirmed with sequencing of samples from non-Japanese populations. Nucleotide transition/transversion ratios in pediatric patients and volunteers are calculated in Supplementary Fig. 5, and are consistent with previous reports^33^ that postulate transition to be much more likely to occur than transversion.

While further investigation will be necessary to make mtDNA sequencing routine, our preprocessing method will facilitate both the sequencing of the entire human mtDNA genome for clinical and forensic purposes and the identification of novel mtDNA SNVs. Despite no evident pathological significance for the novel SNV identified in a healthy volunteer, other disease-associated SNVs were detected in apparently healthy individuals (Supplementary Table 6). As such, our findings will aid in discerning associations between mtDNA mutations and diseases in specific populations (e.g., Japanese) and thereby contribute to the development of molecular diagnostics.

## Supplementary information

SUPPLEMENTARY INFO
