# Supplementary material for: A simple method for sequencing the whole human mitochondrial genome directly from samples and its application to genetic testing
Source: Sci Rep. 2019 Nov 22;9:17411. doi: 10.1038/s41598-019-53449-y (PMC6874554; doi:10.1038/s41598-019-53449-y)
Supplement: Supplementary file 1 — SUPPLEMENTARY INFO [file 41598_2019_53449_MOESM1_ESM.docx]

**A simple method for sequencing the whole human mitochondrial genome directly from samples and its application to genetic testing**

Yue Yao^1+^, Motoi Nishimura^2+^, Kei Murayama^3^, Naomi Kuranobu^3^, Satomi Tojo^1^, Minako Beppu^1,2^, Takayuki Ishige^2^, Sakae Itoga^2^, Sachio Tsuchida^5^, Masato Mori^4^, Masaki Takayanagi^3^, Masataka Yokoyama^1^, Kazuyuki Yamagata^1^, Yoshihito Kishita^5^, Yasushi Okazaki^5^, Fumio Nomura^6^, Kazuyuki Matsushita^2^, Tomoaki Tanaka^1*^

^1^Department of Molecular Diagnosis, Graduate School of Medicine, Chiba University, 1-8-1 Inohana, Chuo-ku, Chiba 260-8670, Japan

^2^Division of Laboratory Medicine, Clinical Genetics and Proteomics, Chiba University Hospital, 1-8-1 Inohana, Chuo-ku, Chiba 260-8670, Japan

^3^Division of Metabolism, Chiba Children's Hospital, Chiba 266-0007, Japan

^4^Department of Pediatrics, Matsudo City Hospital, Matsudo 270-2296, Japan

^5^Diagnostics and Therapeutics of Intractable Diseases, Intractable Disease Research Center, Graduate School of Medicine, Juntendo University, Hongo 2-1-1, Bunkyo-ku, Tokyo, 113-8421, Japan.

^6^Division of Clinical Mass Spectrometry, Chiba University Hospital, 1-8-1 Inohana, Chuo-ku, Chiba 260-8670, Japan

^+^ These authors contributed equally to this study

* Corresponding author

Phone: 81-43-222-7171

Fax: 81-43-226-2095

Email: tomoaki@restaff.chiba-u.jp

**CONFLICT OF INTEREST**

The authors declare no conflict of interest.

**Supplementary Information**





**Supplementary Fig. 1. Electrophoretogram of the products resulting from digestion of amplified mtDNA with EcoRI.**

The EcoRI-mediated cleavage of the peculiar palindrome on the mtDNA (upper panel) generates mtDNA fragments of three sizes (1153 bp, 7366 bp, 8050 bp) that can be visualized using gel electrophoresis (lower panel, with fragments indicated by the red boxes).


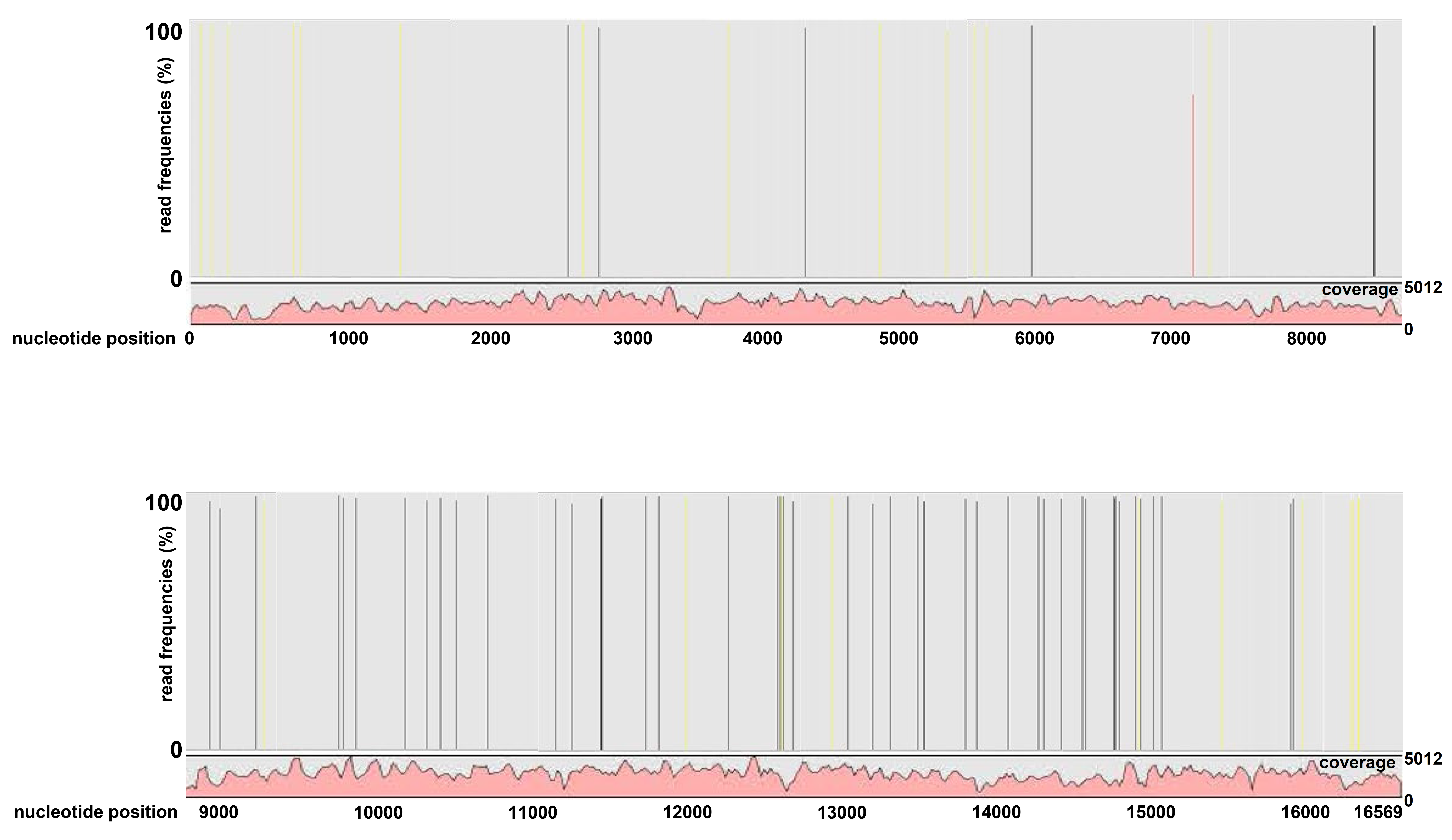


**Supplementary Fig. 2. Frequencies of SNVs found in volunteer A**

SNVs found from 25ng total DNA with read frequencies more than 1% are called are mapped. Homoplasmic SNVs are indicated in yellow, while a heteroplasmic SNV is indicated in red. And a nucleotide position when its read frequencies of major allele is less than 99%, although we judge there is no SNV at that position because of visual inspection of read alignments, are indicated in black. The sequence coverage is reposted from Figure 1.


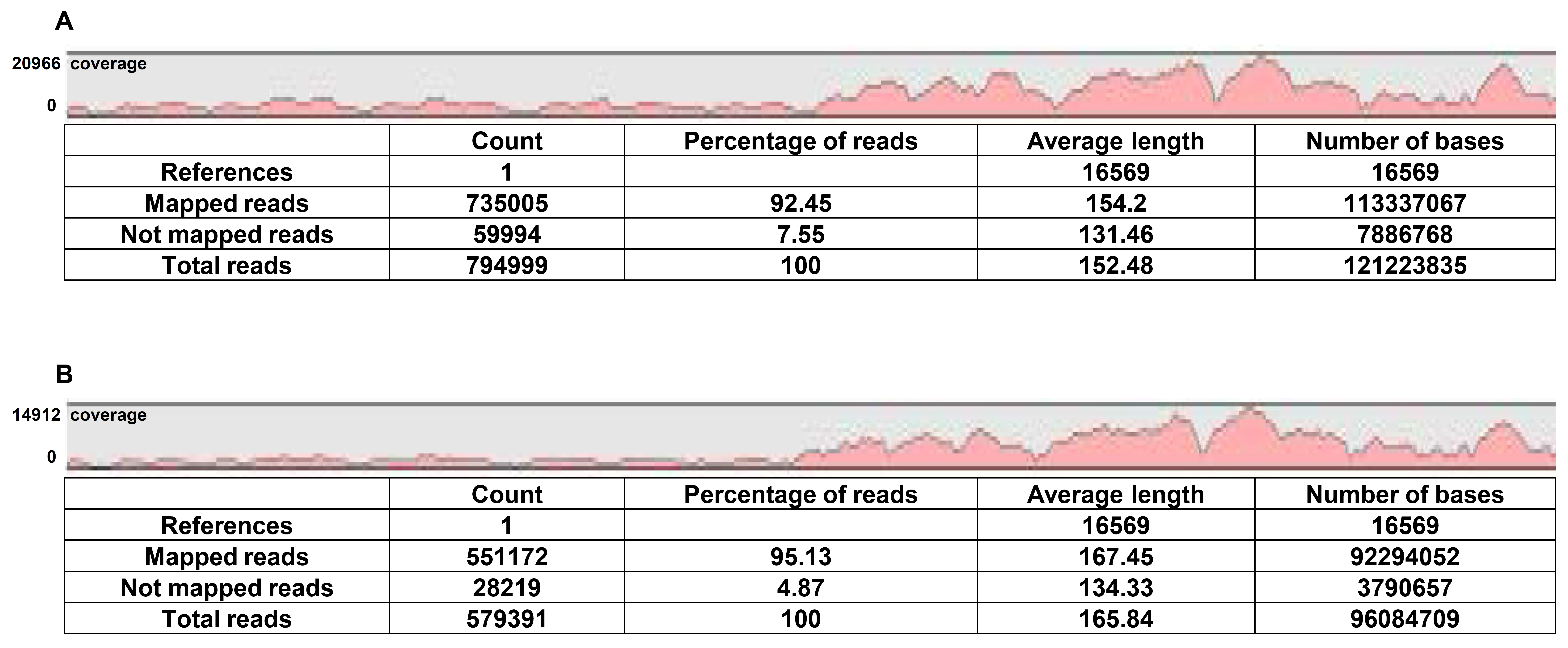


**Supplementary Fig. 3. Mapping and coverage results for whole mtDNA sequencing of plasma samples from volunteers B and C).**

(**A&B**) Sequencing results for mtDNA amplified directly using plasma from volunteer B (**A**) and volunteer C (**B**). The sequencing coverage for each sample is indicated in red. The maximum coverage for mtDNA in volunteer B or C was 20966 reads or 14912 reads, respectively. The results were calculated using CLC Genomics Workbench.


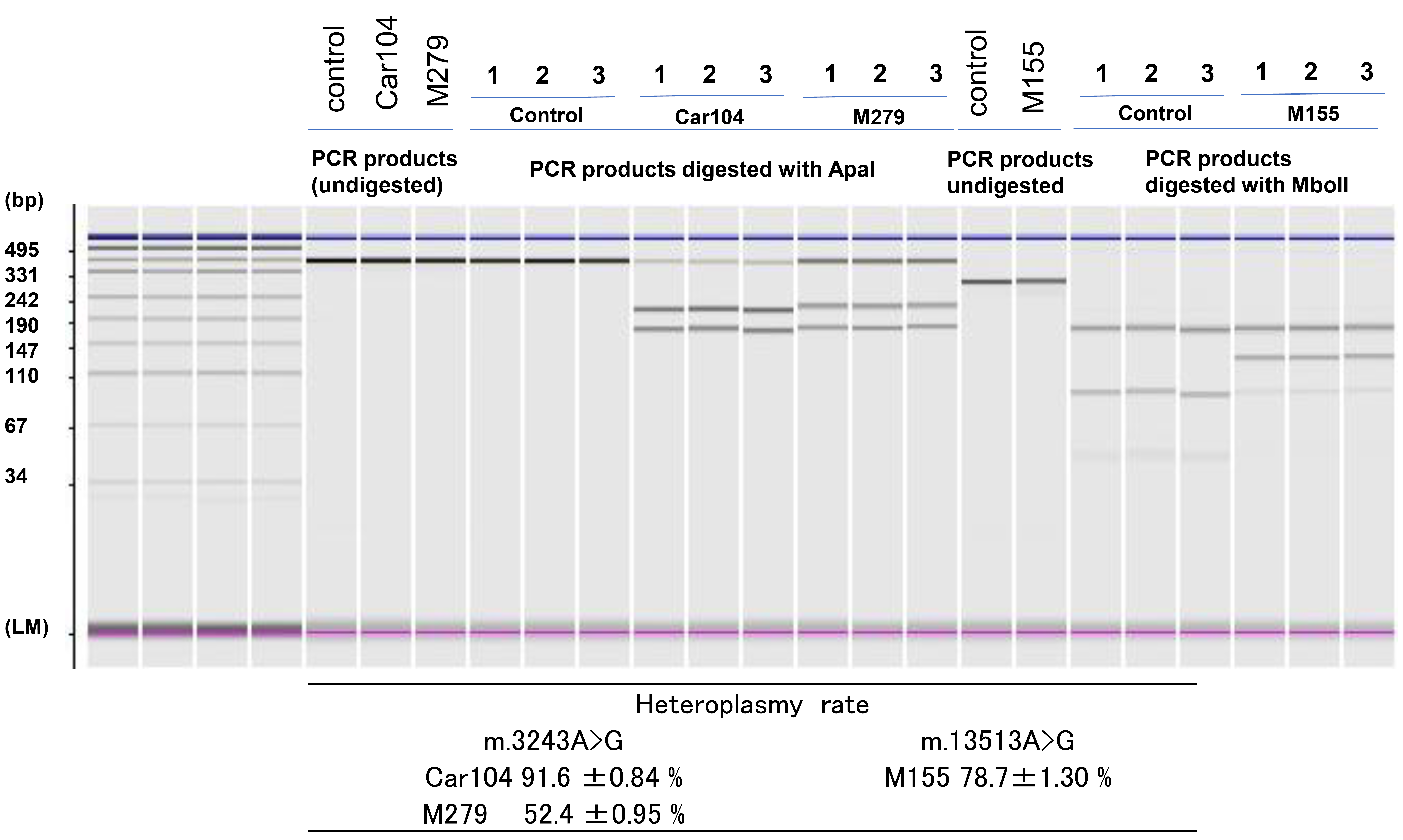


**Supplementary Fig. 4. Heteroplasmy analysis of m.3243A>G and m.13513G>A with PCR-RFLP method**

Heteroplasmy rates of these m.3243A>G and m.13513G>A variations in samples of Car104, M279 and M155 were analyzed by Polymerase Chain Reaction-Restriction Fragment Length Polymorphism (PCR-RFLP) as described previously (*28*) with a modiﬁed protocol. Fragment length and those molar concentration was measured by MultiNA DNA-500 (Shimadzu, Kyoto, Japan). The heteroplasmy rate for m.3243A>G variant was calculated using the following formula from three lanes of digested PCR product: average of digested lower band/(digested lower band + remaining undigested band) and digested upper band/(digested upper band + remaining undigested band) .The proportion of m.13513G>A was calculated by dividing the molar concentration of lower mutant band (MboII-undigested 119 bp) by the molar concentration of upper band (MboII-digested 170 bp). Rates are means±SD of three lanes. These PCR-RFLP analysis were almost consistent with our RCA-based analysis.

**Supplementary Fig. 5.** **mtDNA variations in the volunteers and patients**

mtDNA variations in the volunteers (A) and patients with mutations of single nucleotide substitution (B) are summerized.

In A, nucleotide transition/ transversion ratio is 227/3, while in protein coding region synonymous/ non-synonymous ratio is 97/48. In B, nucleotide transition/ transversion ratio is 183/1, while in protein coding region synonymous/ non-synonymous ratio is 66/45. Single circles indicates variations with nucleotide transition, while double circles indicates variations with nucleotide transversion. Synonymous variations in protein coding region are shown in blue. Non-synonymous variations in protein coding region are shown in red. "(e)" indicates heteroplasmic variation.

| ID | Gender | Sample origin | Disease | Extracted DNA concentration (ng/ul) |
| --- | --- | --- | --- | --- |
| Car104 | F | heart | MELAS | 31.2 |
| M33 | F | muscle | MELAS | 39.5 |
| M52 | M | muscle | Leigh Disease | 24.1 |
| M57 | F | muscle | Leigh Disease | 42.3 |
| M112 | M | muscle | MELAS | 20.8 |
| M132 | F | muscle | Pearson syndrome (bone marrow-pancreatic syndrome) | 47.4 |
| M155 | M | muscle | Leigh Disease | 82.2 |
| M192 | F | muscle | Leigh Disease | 45.3 |
| M166 | F | muscle | Infantile mitochondrial disease | 73.8 |
| M175 | F | muscle | Nicolaides-Baraitser syndrome | 88.8 |
| M249 | M | muscle | Leigh Disease | 76.3 |
| M279 | M | muscle | MELAS | 54.2 |
| M334 | M | muscle | MELAS | 29.6 |
| M528 | F | muscle | MELAS | 43.3 |
| M559 | F | muscle | Leigh Disease | 14.7 |
| Hep575 | M | liver | Pearson syndrome (bone marrow-pancreatic syndrome) | 107.5 |

**Supplementary Table 1. Information for the samples from pediatric patients with mitochondrial disease.**

| volunteer B |  |  |  |
| --- | --- | --- | --- |
| Position | Reference allele | Alternative allele | Heteroplasmy/Homoplasmy |
| 73 | A | G | Homoplasmy |
| 199 | T | C | Homoplasmy |
| 202 | A | G | Homoplasmy |
| 207 | G | A | Homoplasmy |
| 263 | A | G | Homoplasmy |
| 750 | A | G | Homoplasmy |
| 827 | A | G | Homoplasmy |
| 1438 | A | G | Homoplasmy |
| 2706 | A | G | Homoplasmy |
| 2831 | G | A | Homoplasmy |
| 4117 | T | C | Homoplasmy |
| 4769 | A | G | Homoplasmy |
| 4820 | G | A | Homoplasmy |
| 6023 | G | A | Homoplasmy |
| 6413 | T | C | Homoplasmy |
| 7028 | C | T | Homoplasmy |
| 8206 | G | A | Homoplasmy |
| 8860 | A | G | Homoplasmy |
| 11719 | G | A | Homoplasmy |
| 13590 | G | A | Homoplasmy |
| 15236 | A | G | Homoplasmy |
| 16136 | T | C | Homoplasmy |
| 16519 | T | C | Homoplasmy |
| volunteer C |  |  |  |
| Position | Reference allele | Alternative allele | Heteroplasmy/Homoplasmy |
| 73 | A | G | Homoplasmy |
| 185 | G | A | Homoplasmy |
| 263 | A | G | Homoplasmy |
| 750 | A | G | Homoplasmy |
| 1438 | A | G | Homoplasmy |
| 2706 | A | G | Homoplasmy |
| 3244 | G | A | Heteroplasmy |
| 3244 | G | G | Heteroplasmy |
| 4135 | T | C | Homoplasmy |
| 4769 | A | G | Homoplasmy |
| 6386 | C | T | Homoplasmy |
| 7028 | C | T | Homoplasmy |
| 7700 | C | T | Homoplasmy |
| 8275 | C | T | Homoplasmy |
| 8278 | C | A | Homoplasmy |
| 8860 | A | G | Homoplasmy |
| 9123 | G | A | Homoplasmy |
| 10495 | T | C | Homoplasmy |
| 11227 | C | T | Homoplasmy |
| 11719 | G | A | Homoplasmy |
| 14053 | A | G | Homoplasmy |
| 14766 | C | T | Homoplasmy |
| 15326 | A | G | Homoplasmy |
| 15769 | A | G | Homoplasmy |
| 16261 | C | T | Homoplasmy |
| 16299 | A | G | Homoplasmy |
| 16355 | C | T | Homoplasmy |
| 16390 | G | A | Homoplasmy |
| 16519 | T | C | Homoplasmy |

**Supplementary Table 2.** **Summary of the SNVs called from whole mtDNA sequencing analysis of samples from volunteers B and C.** Heteroplasmic positions are shown in red. All variants were called using CLC Genomics Workbench.

| Sample ID | Position | Reference allele | Identified allele | Total  reads | Allele frequency  (%) | Disease |
| --- | --- | --- | --- | --- | --- | --- |
| Car104 | 3243 | A | G | 4159 | 88.74 | MELAS / LS / DMDF / MIDD / SNHL / CPEO / MM / FSGS / ASD / Cardiac+multi-organ dysfunction |
|  |  |  | A |  | 11.26 |  |
| M33 | 12315 | G | A | 5192 | 61.73 | CPEO / KSS |
|  |  |  | G |  | 38.27 |  |
| M155 | 13513 | G | A | 2421 | 75.26 | Leigh Disease / MELAS / LHON-MELAS Overlap Syndrome |
|  |  |  | G |  | 24.74 |  |
| M192 | 14459 | G | A | 1473 | 94.98 | LDYT / Leigh Disease |
|  |  |  | G |  | 5.02 |  |
| M249 | 8993 | T | G | 4470 | 95.57 | NARP / Leigh Disease / MILS / other |
|  |  |  | T |  | 4.43 |  |
| M279 | 3243 | A | G | 4755 | 65.68 | MELAS / LS / DMDF / MIDD / SNHL / CPEO / MM / FSGS / ASD / Cardiac+multi-organ dysfunction |
|  |  |  | A |  | 34.32 |  |

**Supplementary Table 3. Pathogenic mtDNA variants identified in pediatric patients with mitochondrial disease.**

Heteroplasmic positions are shown in red. The data were analyzed using CLC Genomics Workbench and compared with data on Mitomap.

| Sample ID | Position | Reference allele | Alternative allele | Total  reads | Disease |
| --- | --- | --- | --- | --- | --- |
| M166 | 3010 | G | A | 11084 | Cyclic Vomiting Syndrome with Migraine |
|  | 3316 | G | A | 5111 | Diabetes / LHON / PEO |
|  | 8414 | C | T | 1300 | Longevity |
|  | 14668 | C | T | 3292 | Depressive Disorder associated |
|  | 15924 | A | G | 9963 | LIMM |
| M175 | 4833 | A | G | 1510 | Diabetes helper mutation; AD, PD |
|  | 15497 | G | A | 2688 | EXIT / Obesity |
| M279 | 6253 | T | C | 7933 | Prostate Cancer / enriched in POAG cohort |
| M155 | 15662 | A | G | 4097 | Complex mitochondriopathy-associated |
|  | 15927 | G | A | 5580 | Multiple Sclerosis / DEAF1555 increased penetrance / CHD |
| M33 | 4386 | T | C | 4246 | Heart disease / myopathy / hypertension |
|  | 11084 | A | G | 3141 | AD, PD; MELAS |
| M52 | 3394 | T | C | 5357 | LHON / Diabetes / CPT deficiency / high altitude adaptation |
| M57 | 15662 | A | G | 3155 | Complex mitochondriopathy-associated |
|  | 15927 | G | A | 5785 | Multiple Sclerosis / DEAF1555 increased penetrance / CHD |
| M112 | 15662 | A | G | 4106 | Complex mitochondriopathy-associated |
|  | 15927 | G | A | 6485 | Multiple Sclerosis / DEAF1555 increased penetrance / CHD |
| M192 | 1005 | T | C | 2350 | DEAF |
|  | 12338 | T | C | 3350 | DEAF1555 increased penetrance / LHON |
| M528 | 12397 | A | G | 2178 | PD, early onset |
| M559 | 3497 | C | T | 3143 | LHON |
| Hep575 | 663 | A | G | 6752 | Coronary Atherosclerosis risk |
|  | 8794 | C | T | 4961 | Exercise Endurance / Coronary Atherosclerosis risk |

**Supplementary Table 4. mtDNA variants of unknown significance (VUS) and rare polymorphisms identified in pediatric patients with mitochondrial disease.** All variants were homoplasmic.

**Supplementary Table 5. Sequences of the mtDNA control region in 20 healthy volunteers.**

| ID | Position | Reference allele | Alternative allele | Locus | Heteroplasmy/Homoplasmy (Allele frequency) | Disease |
| --- | --- | --- | --- | --- | --- | --- |
| 079C | 11696 | G | A | MT-ND4 | Homoplasmy (100%) | LHON / LDYT / DEAF / hypertension helper mutation |
|  | 7444 | G | A | MT-CO1 | Homoplasmy (100%) | LHON / SNHL / DEAF |
| 080A | 16300 | A | G | MT-CR | Homoplasmy (100%) | BD-associated |
| 082U | 827 | A | G | MT-RNR1 | Homoplasmy (100%) | DEAF |
| 084C | 827 | A | G | MT-RNR1 | Homoplasmy (100%) | DEAF |
|  | 5460 | G | A | MT-ND2 | Homoplasmy (100%) | AD / PD |
| 086Z | 12811 | T | C | MT-ND5 | Homoplasmy (100%) | Possible LHON factor |
| 087X | 16390 | G | A | MT-CR | Homoplasmy (100%) | POAG - potential for association |
| 078Y | 5460 | G | A | MT-ND2 | Homoplasmy (100%) | AD / PD |
|  | 12811 | T | C | MT-ND5 | Homoplasmy (100%) | Possible LHON factor |
| H23S90 | 4833 | A | G | MT-ND2 | Homoplasmy (100%) | Diabetes helper mutation; AD, PD |
| H23S91 | 12372 | G | A | MT-ND5 | Homoplasmy (100%) | Altered brain pH / sCJD patients |
| H23S92 | 4343 | A | G | MT-TQ | Homoplasmy (100%) | Poss. hypertension factor |
| H23S86 | 15951 | A | G | MT-TT | Homoplasmy (100%) | LHON modulator |
| H23S85 | 13708 | G | A | MT-ND5 | Homoplasmy (100%) | LHON / Increased MS risk / higher freq. in PD-ADS |
| 083K | 3644 | T | C | MT-ND1 | Homoplasmy (100%) | BD-associated |

**Supplementary Table 6. Disease-associated SNVs identified by whole mtDNA sequencing of samples from 20 healthy volunteers.** The listed SNVs are registered in the “mtDNA mutations with reports of disease associations” category in Mitomap, and thus variants ranging from ‘likely pathogenic’ to ‘likely benign’ are included.
